# Supplementary material for: UV-B irradiation-activated E3 ligase GmILPA1 modulates gibberellin catabolism to increase plant height in soybean
Source: Nat Commun. 2023 Oct 7;14:6262. doi: 10.1038/s41467-023-41824-3 (PMC10560287; doi:10.1038/s41467-023-41824-3)
Supplement: Supplementary file 1 — Supplementary Information [file 41467_2023_41824_MOESM1_ESM.pdf]

UV-B irradiation-activated E3 ligase GmILPA1 modulates gibberellin catabolism to increase plant height in soybean

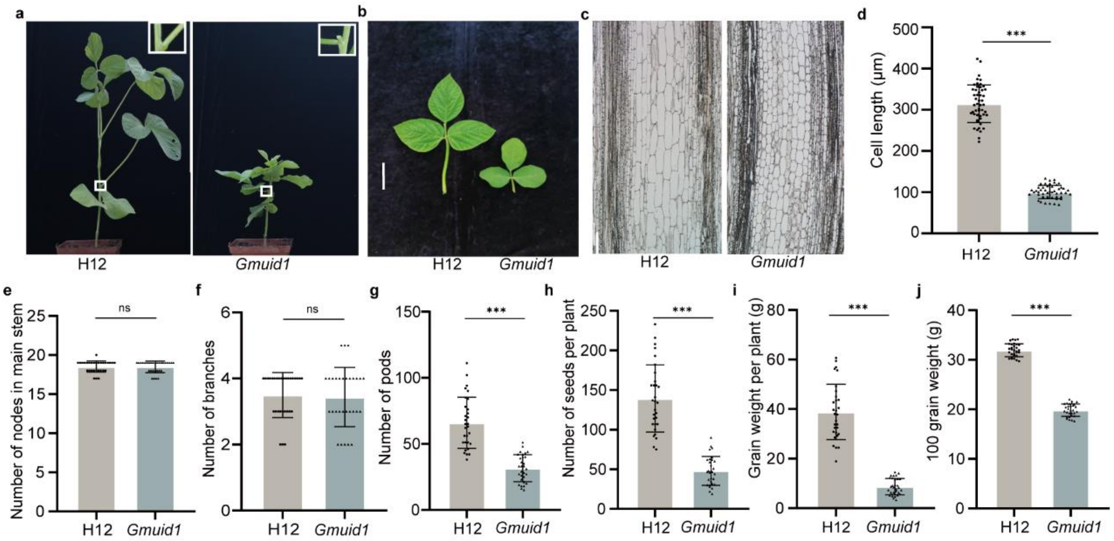

Supplementary Fig. 1 Phenotypes of the wild-type Hedou 12 and the *Gmuid1* mutant

a Leaf petiole angle of Hedou 12 (H12) and *Gmuid1* at the V4 stage (four unrolled trifoliate leaves). The inset shows an enlarged view of leaf petiole angle. **b** Uppermost leaf of H12 and *Gmuid1* at the V4 stage. **c** Longitudinal view of cells from the fifth internode of H12 and *Gmuid1* at the V5 stage (five unrolled trifoliate leaves). **d** Cell length of the fifth internode in H12 and *Gmuid1*. (n = 50 cells from three independent experiments, $P = 2.77\times 10^{-40}$). **e-f** Summary of agronomic traits in H12 and *Gmuid1*. (n = 20 independent plants, $P = 6.28\times 10^{-12}$ in **g**, $P = 1.62\times 10^{-12}$ in **h**, $P = 2.05\times 10^{-20}$ in **i**, $P = 1.41\times 10^{-41}$ in **j**). In **d-j**, Data are presented as mean values +/- SD, Student's *t*-test was used for the significance test, ***$P < 0.001$; ns, not significant. Source data are provided as a Source Data file.

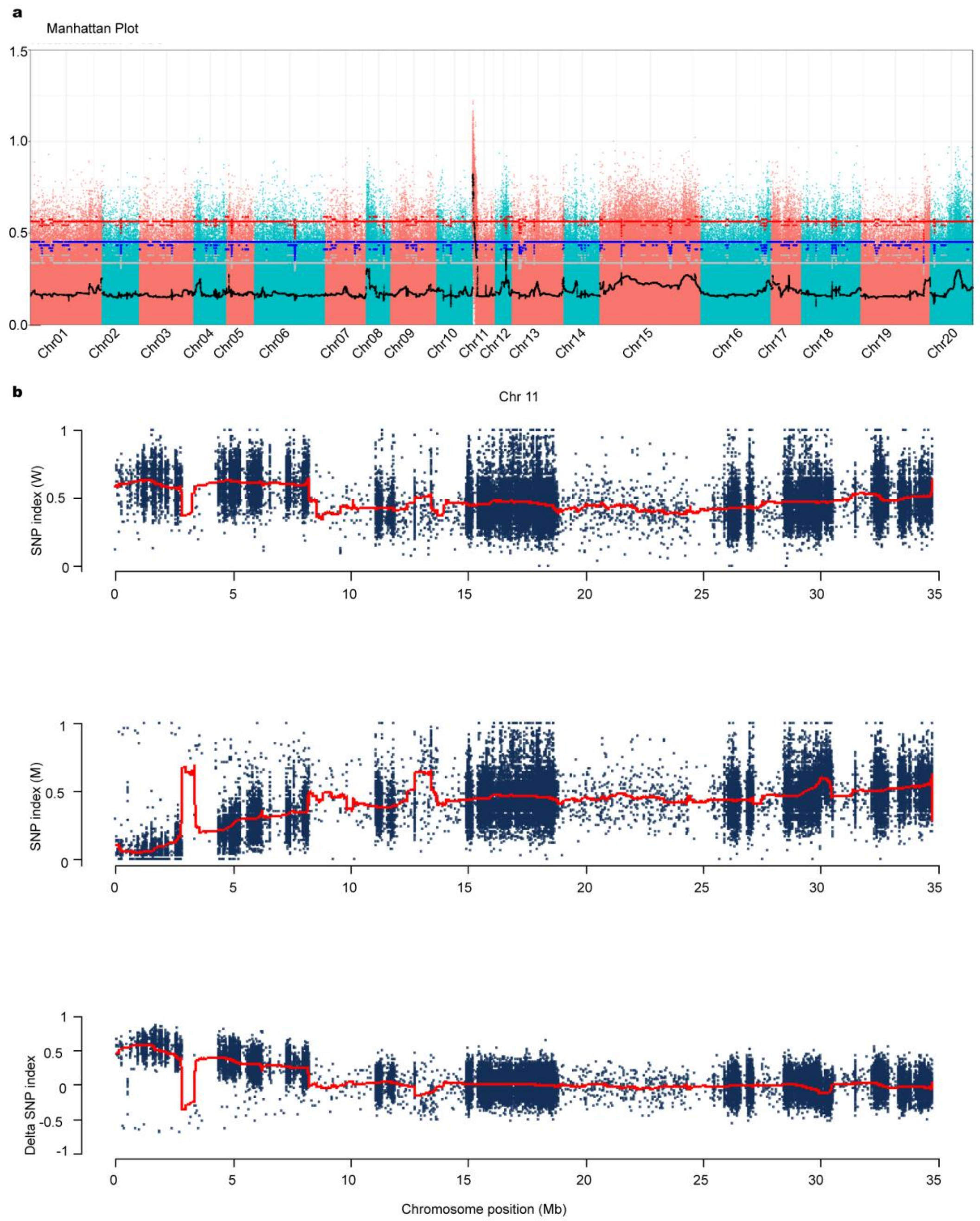

Supplementary Fig. 2 Identification of *GmUID1* by bulked-segregant analysis

a Manhattan Map of Soybean 20 Chromosomes. **b** Mapping-by-sequencing of *Gmuid1*. SNP index ratio for pools of different phenotypic classes (top, wild-type phenotype in Pool W; middle panel, *Gmuid1* phenotype in Pool M). Bottom panel: delta (SNP index) between pool W and pool M, showing the enrichment for Hedou 12 (H12) SNPs at the top of chromosome 11.

```
GmUID1    ATGAGTTCCAAAGAGAGTTGCAGAAGTGAACTTCGCATTGCGATCCGCCAACTCAGTGATCGATGTCTCTACTCTGCTTC
Gmuid1    ATGAGTTCCAAAGAGAGTTGCAGAAGTGAACTTCGCATTGCGATCCGCCAACTCAGTGATCGATGTCTCTACTCTGCTTC
Consensus at gagt t ccaaagagagt t gcagaagt gaact t cgcat t gcgat ccgccaact cagt gat cgat gt ct ct act ct gct t c

GmUID1    TAAATG..........................................................................
Gmuid1    TAAATGGTACCCCTTACAAAAACCCTAATTCAAACAAATTGGGGGTTTCCACTTGACCTCAAGTTTTGAATTTTGAAGTC
Consensus t aaat g

GmUID1    ..................................................................GGCTGCAGAACAGTT
Gmuid1    CCCTTTTACTTTTCTGTGTGATTCCGCAAATGGGTCGTTTGTTTCTGACGAAAATGTGGTTTCAAGGCTGCAGAACAGTT
Consensus                                                                   ggct gcagaacagt t

GmUID1    GGTGGGTATTGAGCAAGACCCTGCCAAGTTCACTCCCTCGAACACGAGATTTCAGCGTGGGAGTTCGAGCATTCGCAGGA
Gmuid1    GGTGGGTATTGAGCAAGACCCTGCCAAGTTCACTCCCTCGAACACGAGATTTCAGCGTGGGAGTTCGAGCATTCGCAGGA
Consensus ggt gggt at t gagcaagaccct gccaagt t cact ccct cgaacacgagat t t cagcgt gggagt t cgagcat t cgcagga

GmUID1    AGTACAAGACTCACGAGATCACGGGAACCCCAATCGCGGGTGTTTCGTATGTTGCCACGCCTGCGATGGAGGAAGATGAG
Gmuid1    AGTACAAGACTCACGAGATCACGGGAACCCCAATCGCGGGTGTTTCGTATGTTGCCACGCCTGCGATGGAGGAAGATGAG
Consensus agt acaagact cacgagat cacgggaaccccaat cgcgggt gt t t cgt at gt t gccacgcct gcgat ggaggaagat gag

GmUID1    CTTGTAGATGGTGATTTCTACCTTCTGGCAAAGTCCTATTTTGATTGCCGTGAGTATAAGAGAGCTGCTCATGTTCTT
Gmuid1    CTTGTAGATGGTGATTTCTACCTTCTGGCAAAGTCCTATTTTGATTGCCGTGAGTATAAGAGAGCTGCTCATGTTCTT
Consensus ct t gt agat ggt gat t t ct acct t ct ggcaaagt cct at t t t gat t gccgt gagt at aagagagct gct cat gt t ct t
```

b

```
GmUID1  MSSKESCRSELRIAIRQLSDRCLYSASKWAAEQLVGIEQDPAKFTPSNTRFQRGSSSIRR
Gmuid1  MSSKESCRSELRIAIRQLSDRCLYSASKWYPLQKP -
GmUID1  KYKTHEITGTPIAGVSYVATPAMEEDELVDGDFYLLAKSYFDCREYKRAAHVLRDQNGRK
Gmuid1
GmUID1  SVFLRCHALYLAGEKRKEEEMIELEGPLGKSDAVNHELVSLERELSTFRKNGKVDPFCL
Gmuid1
GmUID1  YLYGLVLKQKGSENLARAVLVESVNSYPWNWNAWTELQSLCKTVDILNSLNLNSHWMKD
Gmuid1
GmUID1  FFLASVYQELRMHNDSLSKYEYLLGTFSNS NYVQAQIAKAQYSLREFDQVEAIFEELLSN
Gmuid1
GmUID1  DPYRVEDMDMYSNVLYAKECFSALSYLAHRVFMTDKYRPESCCIIGNYYSLKGQHEKSV
Gmuid1
GmUID1  VYFRRALKLNKNFLSAWTLMGHEFVEMKNTPAAVDAYRRAVDIDPRDYRAWYGLGQAYE
Gmuid1
GmUID1  MMGMPFYALHYFKKSVFLQPNDSRLWIAMAQCYETDQLRMLDEAIKCYRRAANCNDREA
Gmuid1
GmUID1  IALHNLAKLHSELGRPEEAAFYYKKDLERMESEEREGPKMVEALLYLAKYYRAQKKFEDA
Gmuid1
GmUID1  EVYCTRLLDYTGPERETAKSILRGMRSTQSNFPSMDVEHFPP-
Gmuid1
```

Supplementary Fig. 3 Sequence alignment between wild-type and mutant *Gmuid1*

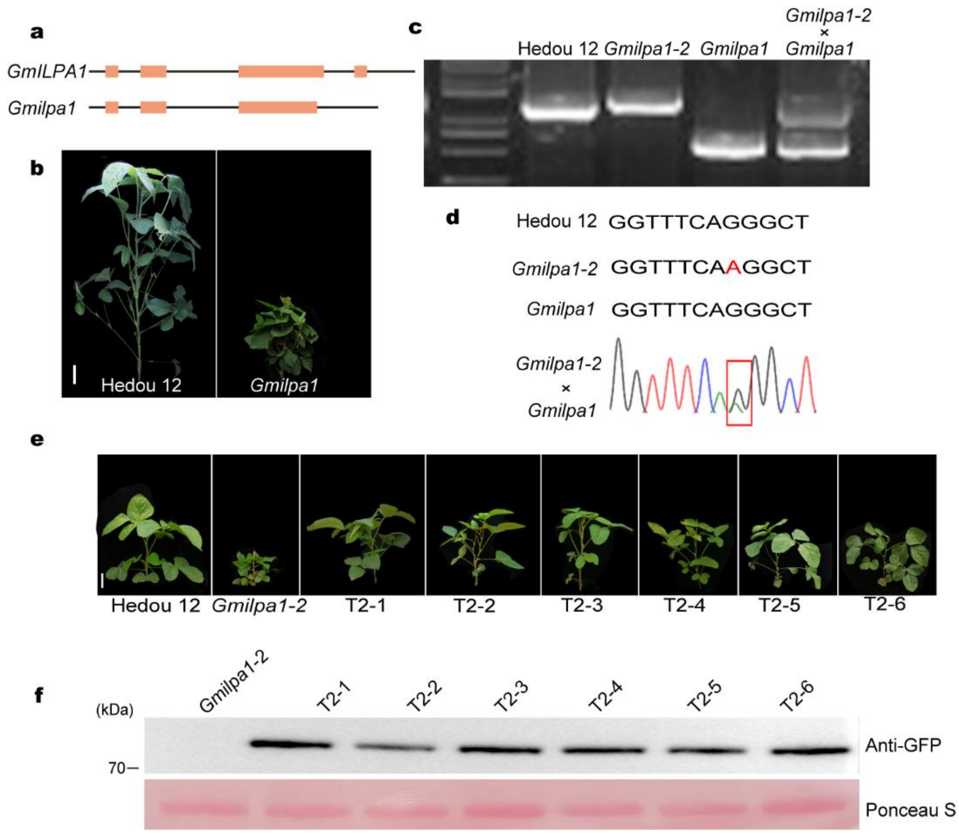

Supplementary Fig. 4 Identification of *GmILPA1*

a-b Schematic diagram of *GmILPA1* in H12 and the *Gmilpa1* mutant, which carries a 1,149-bp deletion that includes 23 bp from intron 3 and exon 4. **c-d** Genomic PCR and sequencing analysis of the *Gmilpa1-2*, *Gmilpa1*, and *Gmilpa1-2* × *Gmilpa1* F1 plants. **e** Representative images of H12, *Gmilpa1-2*, and T2 plants of six transformation events with the *GmILPA1-GFP* transgene. Scale bar, 10 cm. **f** Immunodetection of GFP in the *Gmilpa1-2* mutant and *Gmilpa1-2* T_2 plants expressing the *GmILPA1-GFP* transgene. Source data are provided as a Source Data file.

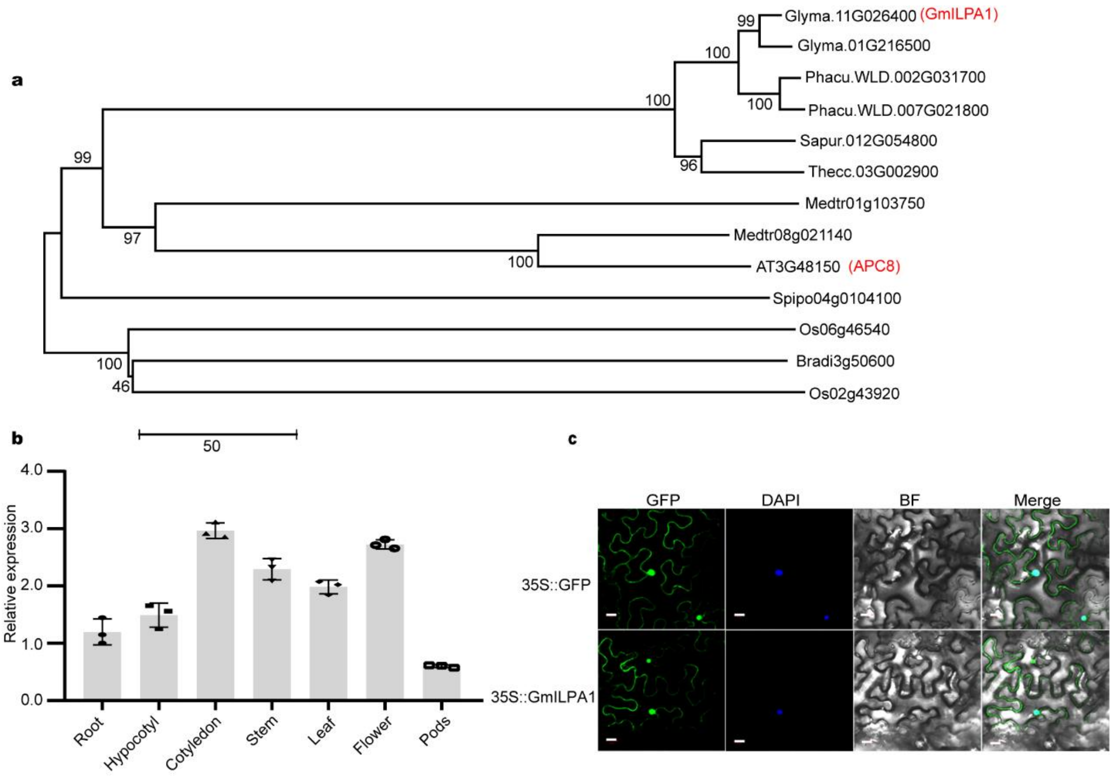

Supplementary Fig. 5 Phylogeny analysis of APC8-like proteins and expression pattern of *GmILPA1*

a Neighbor-joining phylogenetic tree of APC8-like proteins. The tree is based on a multiple protein sequence alignment of the proteins encoded by the following genes: At3g48150 (*Arabidopsis*); Os02g43920 and Os06g46540 (*O. sativa*); Phvul.WLD.002G154200 and Phvul.WLD.007G021500 (*P. vulgaris*); Medtr8g021140 and Medtr1g103750 (*M. truncatula*); Glyma.01G216500 and Glyma.11G026400 (*Glycine max*); Sapur.012G054800 (*Salix purpurea*); Thecc.03G002900 (*Theobroma cacao*); Spipo4G0104100 (*Spirodela polyrhiza*); and Bradi3g50600 (*Brachypodium distachyon*). The numbers at each branch represent the percentage support from 1,000 bootstrap replicates. **b** Relative *GmILPA1* expression levels in different tissues in Hedou12. Data are means ± SD (n = 3). **c** Subcellular localization of GmILPA1-GFP in *N. benthamiana* cells expressing the *35S: GmILPA1-GFP* construct. Scale bar, 50 µm. Source data are provided as a Source Data file.

-

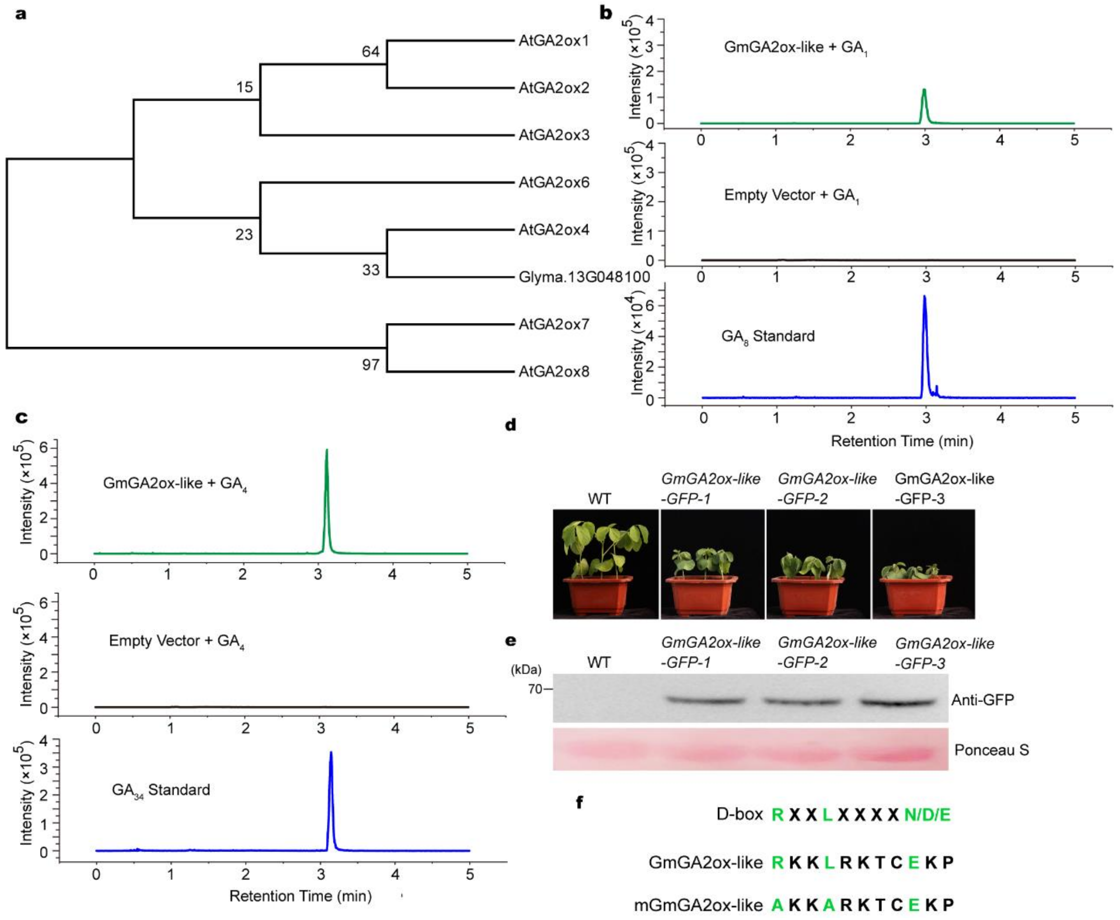

Supplementary Fig. 6 Phylogeny analysis of GmGA2ox-like proteins and identification of transgenic plants

a Neighbour-joining phylogenetic tree of GmGA2ox-like proteins. The tree is based on proteins that encoded the GA2ox in *Arabidopsis*. The numbers at each branch represent the percentage support from 500 bootstrap replicates. **b-c** Conversion of GA_1 to GA_8 (**b**), GA_4 to GA_{34} (**c**) by recombinant GmGA2ox-like protein. Reaction with GST was used as mock. The vertical axis represents the ion signal intensity. **d** Representative images of Hedou 12 (H12) and *GmGA2ox-like-GFP* overexpression lines. **e** Immunodetection of GFP in H12 and *35S:GmGA2ox-like-GFP*. **f** Conserved D-box motif and its mutated construct of GmGA2ox-like. Source data are provided as a Source Data file.

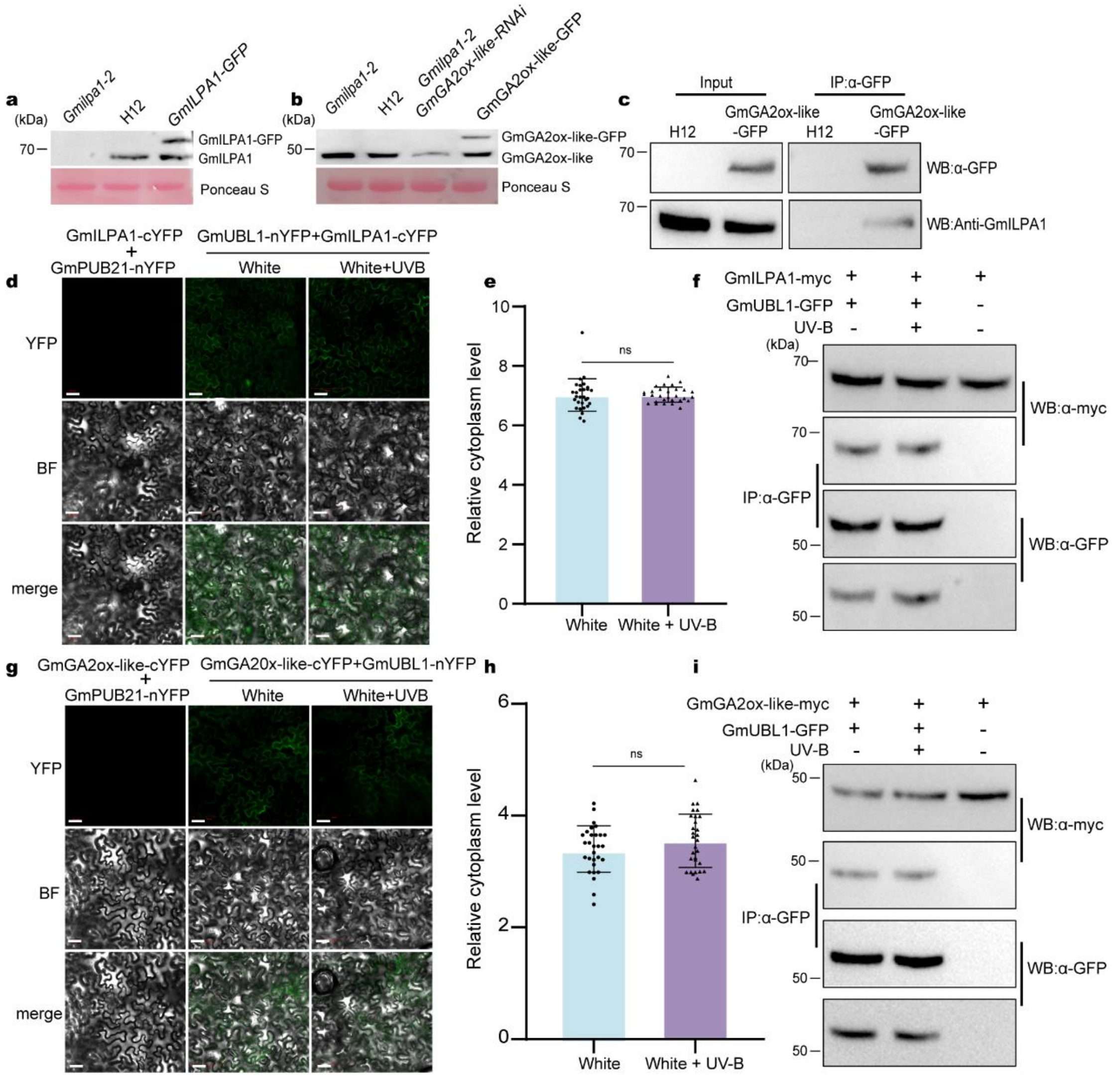

Supplementary Fig. 7 Interaction between GmILPA1 and GmUBL1, GmUBL1, and GmGA2ox-like upon exposure to UV-B light

a Specificity of anti- GmILPA1 antibody. Total proteins extracted from H12, *Gmilpa1-2*, and *GmILPA1-GFP* seedlings were used for immunoblotting analysis with anti-GmILPA1 antibody. **b** Specificity of anti-GmGA2ox-like antibody. Total proteins extracted from H12, *Gmilpa1-2*, *Gmilpa1-2/GmGA2ox-like-RNAi*, and *GmGA2ox-like-GFP* seedlings were used for immunoblotting analysis with anti-GmGA2ox-like antibody. **c** Co-immunoprecipitation assays using seedlings expressing *35S:GmGA2ox-like–GFP*. Immunoprecipitation was performed using GFP-Trap agarose beads, and the immunoblots were probed using anti- GmILPA1 and anti-GFP antibodies. **d** BiFC assays indicating that UV-B treatment did not promote the interaction between GmILPA1 and GmUBL1. Scale bars, 50 µm. **e** Relative YFP fluorescence intensity in the cytoplasm from the images in panel **(d)**. (n = 30 cells, P = 0.9445), the relative fluorescence intensities of cytoplasm and whole cells were quantified and the cytoplasm-to- background ratios are plotted. Data are presented as mean values +/- SD, Student's t-test was used for the significance test, ns, not significant. **f** CoIP assays showed that UV-B did not increase the interaction between GmILPA1 and GmUBL1.

g BiFC assays indicating that UV-B treatment did not promote the interaction between GmGA2ox-like and GmUBL1. Scale bars, 50 μm. **h** (n = 30 cells, *P* = 0.2116), the relative fluorescence intensities of cytoplasm and whole cells were quantified and the cytoplasm-to- background ratios are plotted. Data are presented as mean values +/- SD, Student's *t*-test was used for the significance test, ns, not significant. **i** CoIP assays showed that UV-B did not increase the interaction between GmGA2ox-like and GmUBL1. Source data are provided as a Source Data file.

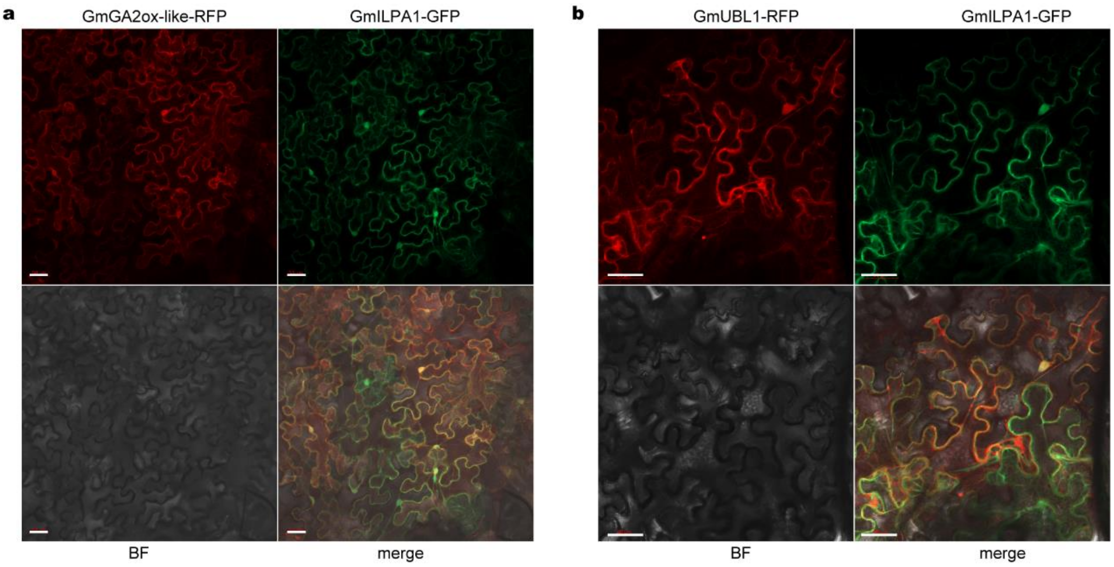

Supplementary Fig. 8 Co-localization of GmILPA1 and GmGA2ox-like, GmILPA1 and GmUBL1

a *N. benthamiana* leaves were co-infiltrated with *GmILPA1-GFP* and *GmGA2ox-like-RFP*. **b** *N. benthamiana* leaves were co-infiltrated with *GmILPA1-GFP* and *GmUBL1-RFP*. Scale bars, 50 μm.

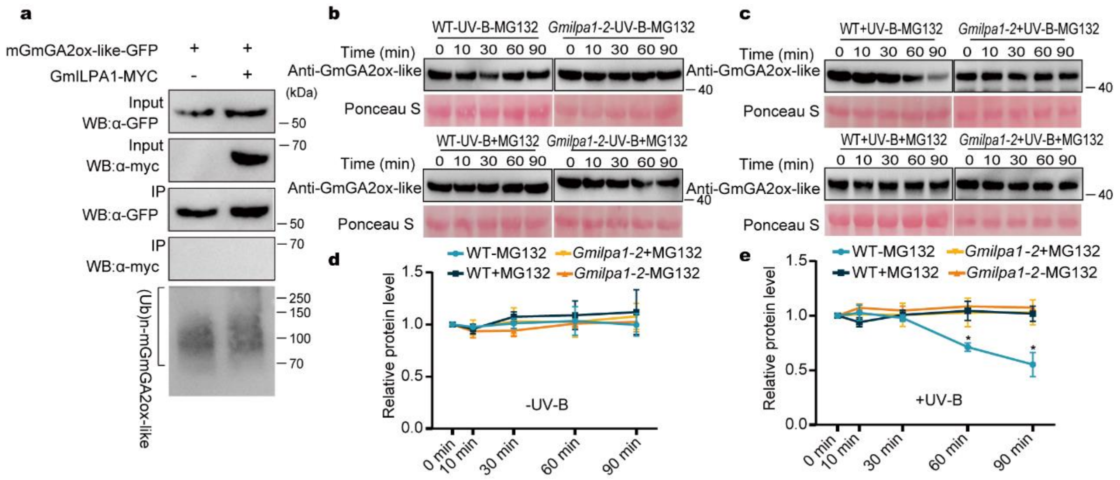

Supplementary Fig. 9 GmILPA1 mediates degradation of GmGA2ox-like

a Ubiquitination of mGmGA2ox-like by GmILPA1. m*GmGA2ox-like-GFP* and *GmILPA1-MYC* were co-infiltrated in *N. benthamiana* leaves. Total protein extracts were immunoprecipitated using anti-GFP antibody-conjugated agarose beads, followed by immunoblotting with anti-ubiquitin antibody. **b-c** GmGA2ox-like degradation in cell-free degradation assays. Total proteins extracted from WT or *Gmilpa1-2* seedlings were incubated with or without MG132 and exposed to UVB or maintained in white light for the indicated time. GmGA2ox-like abundance was detected with anti-GmGA2ox-like antibody and quantified (**d** and **e**). Data are presented as mean values +/- SE, *n* = 3 independent experiments. Source data are provided as a Source Data file.

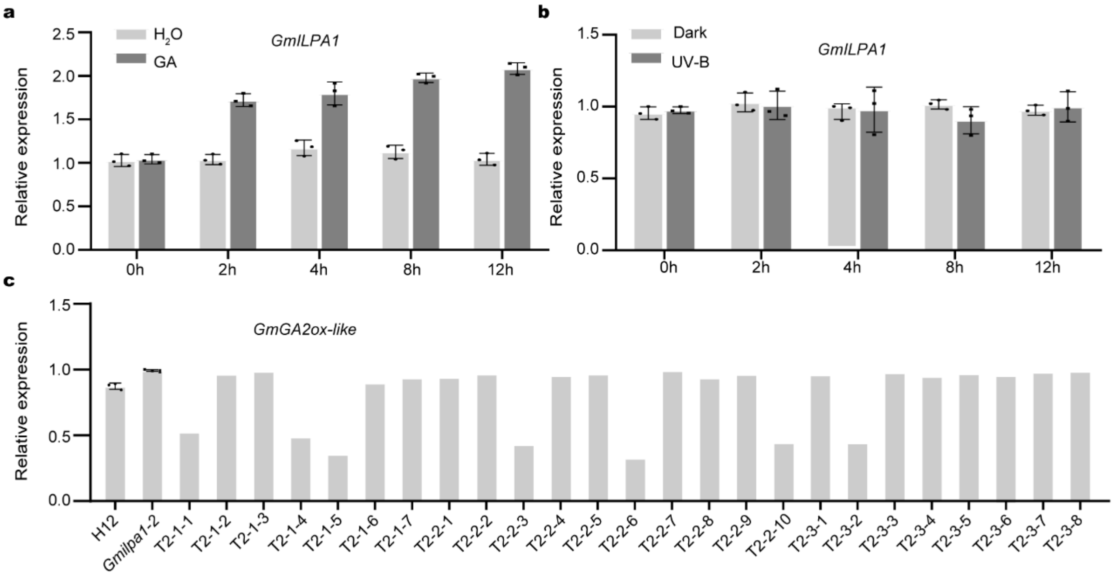

Supplementary Fig. 10 *GmILPA1* transcript levels are differentially regulated by GA_3 and UV-B

a Relative expression levels of *GmILPA1* in Hedou 12 (H12) at V1 stage treated with **exogenous GA_3** as detected by RT-qPCR. **b** Relative expression levels of *GmILPA1* in H12 at the V1 stage exposed to UV-B light as detected by RT-qPCR. Seedlings of H12 grown in darkness for 1 day and then transferred to UV-B for the indicated times. Data are presented as mean values +/- SE, *n* = 3 biologically independent samples. **c** Relative expression levels of *GmGA2ox-like* in Hedou 12 (H12), *Gmilpa1-2* and T2 plants of two transgene lines of *GmGA2ox-like-RNAi* in *Gmilpa1-2*.

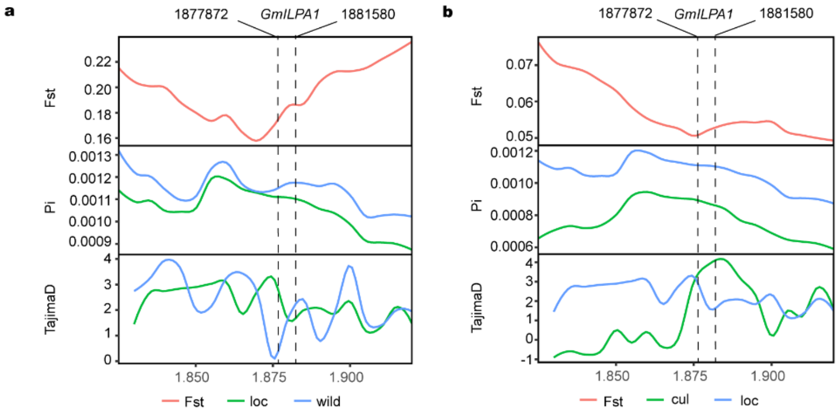

Supplementary Fig. 11 Evolution and geographical distribution of *GmILPA1* haplotypes

a-b FST, nucleotide diversity, and Tajima's D values over an ~100-kb region centered on *GmILPA1* among wild, landrace, and soybean germplasms.

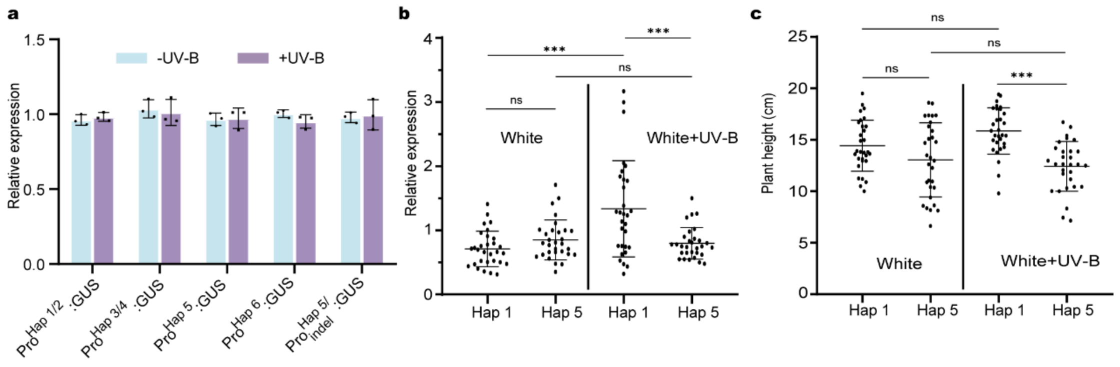

Supplementary Fig. 12 Relative expression of *GmILPA1* in *Hap1* and *Hap5* and plant height in *Hap1* and *Hap5* with and without UV-B exposure

a Relative expression of *Bar* of in *N. benthamiana* leaves infected with GUS vectors with and without UV-B exposure. Data are presented as mean values +/- SD, $n = 3$ biologically independent samples. **b** Relative expression of *GmILPA1* with and without UV-B exposure in *Hap1* and *Hap5*. (n = 30 soybean accessions; $P = 6.94\times10^{-5}$ in white and white + UV-B for Hap 1, $P = 4.36\times10^{-4}$ in Hap 1 and Hap 5 under white + UV-B condition). **c** Height of plants with the or *Hap1* and *Hap5* allele with and without UV-B exposure. (n = 30 soybean accessions; $P = 4.24\times10^{-7}$ in Hap 1 and Hap 5 under white + UV-B condition). Data are presented as mean values +/- SD; *P*-value is calculated with a one-way ANOVA analysis–Tukey comparison, and the columns labeled without the same alphabet are significantly different (P < 0.05, two-sided). ****P* < 0.001, ns, not significant. Source data are provided as a Source Data file.
