# Supplementary material for: UV-B irradiation-activated E3 ligase GmILPA1 modulates gibberellin catabolism to increase plant height in soybean
Source: Nat Commun. 2023 Oct 7;14:6262. doi: 10.1038/s41467-023-41824-3 (PMC10560287; doi:10.1038/s41467-023-41824-3)
Supplement: Supplementary file 3 — Description of Additional Supplementary Files [file 41467_2023_41824_MOESM3_ESM.pdf]

Description of Additional Supplementary Files

File Name: Supplementary Data 1
Description: Ion’s detection information for GA content determination

File Name: Supplementary Data 2
Description: Primers used in this study
