# Supplementary material for: UV-B irradiation-activated E3 ligase GmILPA1 modulates gibberellin catabolism to increase plant height in soybean
Source: Nat Commun. 2023 Oct 7;14:6262. doi: 10.1038/s41467-023-41824-3 (PMC10560287; doi:10.1038/s41467-023-41824-3)
Supplement: Supplementary file 6 — Reporting Summary [file 41467_2023_41824_MOESM6_ESM.pdf]

nature portfolio

Corresponding author(s): Fengning Xiang; Fanjiang Kong; Baohui Liu; Xianzhong Feng

Last updated by author(s): Aug 31, 2023

Reporting Summary

Nature Portfolio wishes to improve the reproducibility of the work that we publish. This form provides structure for consistency and transparency in reporting. For further information on Nature Portfolio policies, see our Editorial Policies and the Editorial Policy Checklist.

Statistics

For all statistical analyses, confirm that the following items are present in the figure legend, table legend, main text, or Methods section.

n/a	Confirmed	
☐	☒	The exact sample size (*n*) for each experimental group/condition, given as a discrete number and unit of measurement
☐	☒	A statement on whether measurements were taken from distinct samples or whether the same sample was measured repeatedly
☐	☒	The statistical test(s) used AND whether they are one- or two-sided<br>*Only common tests should be described solely by name; describe more complex techniques in the Methods section.*
☒	☐	A description of all covariates tested
☒	☐	A description of any assumptions or corrections, such as tests of normality and adjustment for multiple comparisons
☐	☒	A full description of the statistical parameters including central tendency (e.g. means) or other basic estimates (e.g. regression coefficient) AND variation (e.g. standard deviation) or associated estimates of uncertainty (e.g. confidence intervals)
☐	☒	For null hypothesis testing, the test statistic (e.g. *F*, *t*, *r*) with confidence intervals, effect sizes, degrees of freedom and *P* value noted<br>*Give P values as exact values whenever suitable.*
☒	☐	For Bayesian analysis, information on the choice of priors and Markov chain Monte Carlo settings
☒	☐	For hierarchical and complex designs, identification of the appropriate level for tests and full reporting of outcomes
☒	☐	Estimates of effect sizes (e.g. Cohen's *d*, Pearson's *r*), indicating how they were calculated

Our web collection on statistics for biologists contains articles on many of the points above.

Software and code

Policy information about availability of computer code

Data collection	The SNP/Indel data of soyben accessions (Nat Genet 49, 773–779 (2017)) were used in this study.
Data analysis	The plant height, the fluorescent signal intensity and the relative fold change of western blot band were measured by Image J software. Confocal microscopy was performed using the LSM-700laser scanning confocal microscope(Zeiss). Haplotype network analysis was performed using PopART software. Phylogenetic analysis was based on UPGMA tree implemented in MEGA7 software. Quantitative data were subjected to statistical analysis performed with the GraphPad Prism 8.0.

For manuscripts utilizing custom algorithms or software that are central to the research but not yet described in published literature, software must be made available to editors and reviewers. We strongly encourage code deposition in a community repository (e.g. GitHub). See the Nature Portfolio guidelines for submitting code & software for further information.

Data

Policy information about availability of data

All manuscripts must include a data availability statement. This statement should provide the following information, where applicable:

- Accession codes, unique identifiers, or web links for publicly available datasets
- A description of any restrictions on data availability
- For clinical datasets or third party data, please ensure that the statement adheres to our policy

The Williams 82 soybean reference genome sequence was downloaded from the Phytozome v12 (https://phytozome.jgi.doe.gov/pz/

portal.html). The SNP/Indel data of natural population accessions were previously reported were deposited into the NCBI database under accession number PRJNA394629 and the Genome Sequence Archive database in BIG Data Center under accession numbers PRJCA000205 and PRJCA001691.

Research involving human participants, their data, or biological material

Policy information about studies with human participants or human data. See also policy information about sex, gender (identity/presentation), and sexual orientation and race, ethnicity and racism.

Reporting on sex and gender	N/A
Reporting on race, ethnicity, or other socially relevant groupings	N/A
Population characteristics	N/A
Recruitment	N/A
Ethics oversight	N/A

Note that full information on the approval of the study protocol must also be provided in the manuscript.

Field-specific reporting

Please select the one below that is the best fit for your research. If you are not sure, read the appropriate sections before making your selection.

☒ Life sciences ☐ Behavioural & social sciences ☐ Ecological, evolutionary & environmental sciences

For a reference copy of the document with all sections, see nature.com/documents/nr-reporting-summary-flat.pdf

Life sciences study design

All studies must disclose on these points even when the disclosure is negative.

Sample size	The samples for mRNA expression, cell-free protein abundance, plant height measurement, determination of gibberellin content were analyzed at least with three biological replicates. Cytoplasmic fuorescence signals of BiFC from more than 30 cells were measured using ImageJ software. Sample sizes are indicated in the main text, figures and legends. We did not use any statistical method to predetermine sample size.
Data exclusions	No data were excluded from our analyses.
Replication	All experiments were independently performed third and result were successfully repeated.
Randomization	All samples were arranged randomly into experimental groups.
Blinding	All the experiments were performed without prior knowledge of the final outcome, and therefore blinding was not applied.

Reporting for specific materials, systems and methods

We require information from authors about some types of materials, experimental systems and methods used in many studies. Here, indicate whether each material, system or method listed is relevant to your study. If you are not sure if a list item applies to your research, read the appropriate section before selecting a response.

Materials & experimental systems

n/a	Involved in the study	
☐	☒	Antibodies
☐	☒	Eukaryotic cell lines
☒	☐	Palaeontology and archaeology
☒	☐	Animals and other organisms
☒	☐	Clinical data
☒	☐	Dual use research of concern
☒	☐	Plants

Methods

n/a	Involved in the study	
☒	☐	ChIP-seq
☒	☐	Flow cytometry
☒	☐	MRI-based neuroimaging

Antibodies

Antibodies used

Mouse monoclonal anti GFP (Proteintech, Wuhan, China; Cat No: 66002-1-Ig;1:5000)
Mouse monoclonal anti MYC (Proteintech, Wuhan, China; Cat No: 60003-2-Ig;1:5000)
Mouse monoclonal anti Flag (Proteintech, Wuhan, China; Cat No:66008-4-Ig;1:1000)
Rabbit ubiquitin polyclonal antibody (Proteintech, Wuhan, China; Cat No:10201-2-AP;1:1000)
Mouse monoclonal anti MBP (Proteintech, Wuhan, China; Cat No:66003-1-Ig;1:5000)
Secondary antibody; HRP-conjugated Affinipure Goat Anti-Rabbit IgG(H+L) (Proteintech, Wuhan, China; Cat No:SA00001-2;1:5000)
Secondary antibody; HRP-conjugated Affinipure Goat Anti-Mouse IgG(H+L)(Proteintech, Wuhan, China; Cat No:SA00001-1;1:5000)

Validation

All antibodies are commercially available and widely used in the scientific community. The following antibodies were validated by the supplier:
Mouse monoclonal anti GFP: https://www.ptgcn.com/products/eGFP-Antibody-66002-1-Ig.htm
Mouse monoclonal anti MYC: https://www.ptgcn.com/products/MYC-Antibody-60003-2-Ig.htm
Mouse monoclonal anti Flag:https://www.ptgcn.com/products/Flag-tag-Antibody-66008-4-Ig.htm
Rabbit ubiquitin polyclonal antibody:https://www.ptgcn.com/products/ubiquitin-Antibody-10201-2-AP.htm
Mouse monoclonal anti MBP:https://www.ptgcn.com/products/MBP-Tag-Antibody-66003-1-Ig.htm
HRP-conjugated Affinipure Goat Anti-Rabbit IgG(H+L):https://www.ptgcn.com/products/HRP-conjugated-Affinipure-Goat-Anti-Rabbit-IgG-H-L-secondary-antibody.htm
HRP-conjugated Affinipure Goat Anti-Mouse IgG(H+L)：https://www.ptgcn.com/products/HRP-conjugated-Affinipure-Goat-Anti-Mouse-IgG-H-L-secondary-antibody.htm

Eukaryotic cell lines

Policy information about cell lines and Sex and Gender in Research

Cell line source(s)

Y2H gold is a strain of yeast used in biological research for two hybrid screening.

Authentication

The plasmid was transformed into the yeast strain Y2H gold using the lithium acetate method.

Mycoplasma contamination

Y2H gold cell line was negative for mycoplasma contamination.

Commonly misidentified lines
(See ICLAC register)

No cell lines used were listed in the database of commonly misidentified cell lines.
